# ﻿Four new species and two newly recorded species of Limacodidae (Lepidoptera, Zygaenoidea) from China

**DOI:** 10.3897/zookeys.1123.77217

**Published:** 2022-10-07

**Authors:** Jun Wu, Alexey V. Solovyev, Hui-Lin Han

**Affiliations:** 1 School of Forestry, Northeast Forestry University, Harbin, 150040, China Northeast Forestry University Harbin China; 2 Department of Biology and Chemistry, Ulyanovsk State Pedagogical University, Ulyanovsk, 432071, Russia Ulyanovsk State Pedagogical University Ulyanovsk Russia; 3 Northeast Asia Biodiversity Research Center, Northeast Forestry University, Harbin, 150040, China Northeast Forestry University Harbin China; 4 Key Laboratory of Sustainable Forest Ecosystem Management, Ministry of Education, Northeast Forestry University, Harbin, 150040, China Ulyanovsk State Pedagogical University Ulyanovsk Russia

**Keywords:** Checklist, new record, slug caterpillar moth, southwest China, taxonomy, Xizang, Yunnan

## Abstract

Four new species, *Kitanolashilinensis***sp. nov.**, *K.eleganta***sp. nov.**, *Fignyaravalba***sp. nov.**, and *Euphlyctinidespseudolaika***sp. nov.**, are described from southwestern China. Two species are reported new to China, *Euphlyctinidisindi* Solovyev, 2009 and *Limacocerapachycera* (Hampson, 1897). The adults and genitalia of all the treated species are illustrated. A checklist for the species belonging to the treated genera is provided.

## ﻿Introduction

The family Limacodidae, more commonly known as slug caterpillar moths, contains 301 genera and 1672 species globally (van Nieukerken et al. 2011). Wu et al. (2022) estimated the number of slug moths is nearly, or already more than, 1750 species to date. The diversity of Chinese Limacodidae, especially in southern China, is rich but poorly studied. Wu (2010) reported 64 genera and 230 species in China, including 89 species with larval host plant records.

This study aims to describe four new species and to report two unrecorded species in the family Limacodidae from the Xizang Autonomous Region (= Tibet) and Yunnan Province, southwest China. These species belong to the genera *Fignya* Solovyev & Witt, 2009, *Kitanola* Matsumura, 1925, *Euphlyctinides* Hering, 1931, and *Limacocera* Hering, 1931. Brief introductions to these genera are given below.

## ﻿Materials and methods

The specimens were collected in field, using a 220 V/450 W mercury light and a DC black light. Wingspan was measured from forewing apex to apex. Standard methods were used to dissect and prepare slides of the genitalia (Kononenko and Han 2007). The specimens were photographed using a Nikon D700 camera, and the genitalia slides were photographed using an Olympus photo microscope aided by Helicon Focus software and further processed using Adobe Photoshop CS6.

All the specimens examined, including the type specimens, were deposited in the collection of the Northeast Forestry University (**NEFU**), Harbin, China. The specimens for comparison were borrowed from the Museum Witt München/Zoologische Staatssammlung München, Munich, Germany (**MWM/ZSM**), the Zoological Institute of Russian Academy of Sciences, St. Petersburg, Russia (**ZISP**), and the collection of Alexey V. Solovyev, Ulyanovsk, Russia (**CASU**).

## ﻿Taxonomic account

### ﻿Genus Kitanola

Taxon classificationAnimaliaLepidopteraLimacodidae

Matsumura, 1925

CE9EEDCE-CF3A-5364-B3D2-69A1CA868559

Kitanola Matsumura, 1925: 116. Type species: Kitanolasachalinensis Matsumura, 1925.
Microcampa Kawada, 1930: 256. Type species: Heterogenauncula Staudinger, 1887.
Mediocampa Inoue, 1982: 301. Type species: Kitanolaspeciosa Inoue, 1956.

#### Note.

Members of the genus *Kitanola* Matsumura, 1925 are small in size. *Kitanola* species have up-curved labial palpi, filiform male antennae, and forewing veins R_3_+R_4_ stalked with R_5_. The tibial spurs are 0-2-4. The uncus and gnathos in the male genitalia are usually or slightly widened, and the transtilla usually bears a long process. The genus is mainly distributed in eastern Asia and contains 10 species to date, eight of which are recorded from China (Inoue 1956; Tshistjakov 1995; Sasaki 1998; Solovyev 2008; Wu and Fang 2008; Hirowatari et al. 2013).

### ﻿ Kitanola shilinensis sp. nov.

Taxon classificationAnimaliaLepidopteraLimacodidae

A0F8EADC-8019-5BA0-A20A-72C51904CFB3

https://zoobank.org/464D8EB1-4EF1-4398-A096-494FA5AC4A06

Figs 1 , 15

#### Material examined.

***Holotype*.** ♂, China, Prov. Yunnan, Kunming City, Shilin County, Changhu Town, Changhu wetland park, 23–28.VIII.2020, KL. Wu leg., genit. prep. WuJ-248-1 (NEFU). ***Paratypes*.** 2♂, China, same data as for holotype, genit. prep. WuJ-247-1, WuJ-299-1 (NEFU).

#### Diagnosis.

**Figures 1–6.**
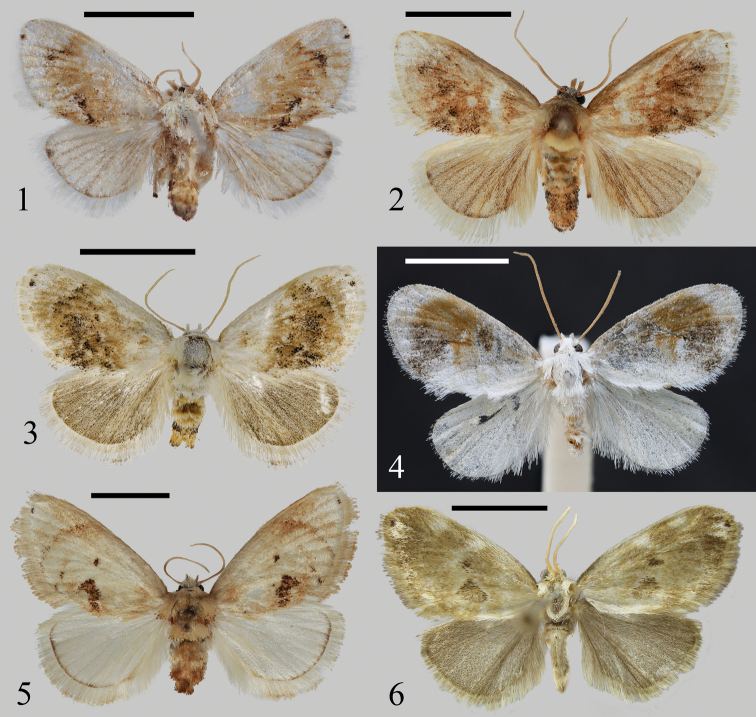
Adults of *Kitanola* spp. **1***K.shilinensis* sp. nov., holotype, Yunnan, China (NEFU) **2***K.spina* Wu & Fang, 2008, Chongqing, China (NEFU) **3***K.spinula* Wu & Fang, 2008, Zhejiang, China (NEFU) **4***K.eleganta* sp. nov., holotype, Xizang, China (NEFU) **5***K.linea* Wu & Fang, 2008, Guangdong, China (NEFU) **6***K.uncula* (Staudinger, 1887), Sakhalin, Russia (ZISP). Scale bars: 5 mm.

The new species *K.shilinensis* sp. nov. (Fig. 1) is hardly separable from *K.spina* Wu & Fang, 2008 (Fig. 2) and *K.spinula* Wu & Fang, 2008 (Fig. 3), but there are several distinguishing features in the male genitalia, as follows (the details of the latter two species are in parentheses): the serrated transtilla is small with only one single long finger-shaped process on its lateral side in *K.shilinensis* sp. nov. (Fig. 15), whereas in *K.spina* the smooth transtilla has a thick finger-shaped process (Fig. 16) and in *K.spinula* (Fig. 17) the serrated transtilla is larger and has two lateral processes (one long, the other short) compared to *K.shilinensis* sp. nov.; the terminal part of aedeagus with two groups of strongly sclerotized spines in *K.shilinensis* sp. nov. (the terminal part of aedeagus with a circle of strongly sclerotized spines in *K.spina*; the terminal part of aedeagus with a cluster of fine spines in *K.spinula*).

#### Description.

Adult (Fig. 1). Forewing length 7.0–7.5 mm, wingspan 14.5–15.0 mm. Head yellowish white; labial palpus up-curved; antennae filiform, brown. Thorax yellowish white. Forewing ground colour yellowish white, covered with dense brown scales, with a large brown patch in medium part; M-area and inner margin area covered with black scales; outer margin with two distinct black dots near apex; fringe long, greyish white. Hindwing pale brown, with a distinct black dot near apex; fringe greyish white. Abdomen brown, dark brown terminally. Scales on legs greyish white, terminal of tarsus black.

***Male genitalia*** (Fig. 15). Both lateral processes of uncus broad, densely covered with short hairs, with a very small apical spur. Gnathos short, acute apically. Valva slender, narrow at base, medial part with a sclerotized region near the sacculus process; cucullus visibly narrowing at lower part; transtilla broad, strongly sclerotized, posterior margin serrated, with a long finger-shaped process on lateral side; sacculus narrow, slightly inflated at base; sacculus process strongly sclerotized, small triangular in shape. Aedeagus slender, usually with two groups (each with 1–3) strongly sclerotized, robust spines at the terminal.

**Female.** Unknown.

#### Bionomics.

The specimens were collected in late August at altitudes of 1,850 m a.s.l. The collection site was a wetland park, surrounded mainly by planted pine (family Pinaceae) and camphor (family Lauraceae) trees and some landscaping vegetation, with a large number of grasses growing as a ground cover layer in the woods (Fig. 32).

#### Distribution

**(Fig. 29).** China (Yunnan).

#### Etymology.

The species is named *shilinensis* after its type locality in Shilin County, Yunnan Province, China.

### ﻿ Kitanola eleganta sp. nov.

Taxon classificationAnimaliaLepidopteraLimacodidae

B06A1A9E-64B7-5695-BF8B-C4C400530CE9

https://zoobank.org/E5445A39-A21F-41D5-89AD-F0778EE5D949

Figs 4 , 18

#### Material examined.

***Holotype*.** ♂, China, Xizang Autonomous Region, Linzhi (= Nyingchi) City, Motuo (= Medog) County, Gedang Countryside, 25–30.V.2021, J. Wu and JJ. Fan legs (NEFU). ***Paratypes*.** 2♂, China, same data as for holotype, genit. prep. WuJ-500-1, WuJ-501-1 (NEFU).

#### Diagnosis.

The new species (Fig. 4) is somewhat similar in appearance to *K.shilinensis* sp. nov. (Fig. 1), *K.spina* (Fig. 2), and *K.spinula* (Fig. 3), but it can be distinguished from these by the ground colour of the forewing and thorax, which is white; the forewing with a large patch, which is composed of brown and dark brown; the hindwing is white; and the abdomen is brown alternating with white. In contrast, in the three similar species, the ground colour of the forewing and thorax is yellowish white; the forewing has a broad, dark yellowish-brown band; the hindwings are greyish brown to brown; and the abdomen is brown to dark brown.

It can be also separated from these three species by the following male genitalia characters. In *K.eleganta* sp. nov. (Fig. 18), the uncus is acute apically; the transtilla is lacking; the valva bears a conspicuous triangular basal spine on costa and a strongly sclerotized, eagle-claw-shaped process near middle of sacculus; the aedeagus is short, has an apically bifid, long spur. However, in *K.shilinensis* sp. nov. (Fig. 15), *K.spina* (Fig. 16), and *K.spinula* (Fig. 17), the uncus is broad; the transtilla is present (in *K.spina* the serrated transtilla is lacking a thick finger-shaped lateral process is present); the aedeagus is slender, with the various numbers of apical spines or spinules.

*Kitanolaeleganta* sp. nov. differs markedly in appearance from *K.linea* Wu & Fang, 2008 (Fig. 5) and *K.uncula* (Staudinger, 1887) (Fig. 6) mainly in that the new species has a white ground colour and lacks a small black spot near the apex of the forewing, whereas the latter two are greyish white to ochreous in ground colour and usually have a small black spot near the apex. However, in the male genitalia, the new species has more similar to *K.linea* (Fig. 19) and *K.uncula* (Fig. 20), but it can be distinguished by the following characters: in *K.eleganta* sp. nov., the uncus is small, the sacculus bears an eagle-claw-shaped process, and the aedeagus is short, with a long bifid spur terminally; in *K.linea* and *K.uncula*, the uncus is large, the process located in the sacculus is straight, and the aedeagus is sinuous and with a large apical spine.

#### Description.

Adult (Fig. 4). Forewing length 9.0–9.5 mm, wingspan 18–20 mm. Head white; labial palpus up-curved, brown; antennae filiform, brown. Thorax white. Forewing ground colour white, covered with sparse dark brown scales; smoothly curved subterminal line runs from the costal margin near apex to tornus, terminal area crescent-like, white, tinted slightly brown; inner margin area white; rest mainly with large irregular brown and dark brown patches; fringe white to dark brown. Hindwing ground colour white with M-area tinted pale brown. Abdomen brown alternating with white, terminal white.

***Male genitalia*** (Fig. 18). Uncus and gnathos slender, pointed apically. Basal half of valva without setae, whereas upper half densely covered with setae; valva with a conspicuous triangular spine on the base of costa and a strongly sclerotized, eagle-claw-shaped process near middle of sacculus, with six or seven strongly sclerotized, slightly curved spines on the outer margin; cucullus narrow and rounded; sacculus slightly sclerotized at base; sacculus process not obvious, showing as a hairy rounded papula. Juxta flattened, nearly square. Saccus conspicuous, broadly tongue-shaped. Aedeagus short, caecum large, tapering towards apex; terminal part with a strongly sclerotized, bifid apically spur that almost same length as aedeagus.

**Female.** Unknown.

#### Bionomics.

The specimens were collected in May at an altitude of 2,120 m a.s.l., near a subtropical evergreen broadleaf forest, with massive shrubs, ferns, and patches of grassland growing as the ground cover layer in the forest (Fig. 31).

#### Distribution

**(Fig. 29).** China (Xizang).

#### Etymology.

The species name, a noun in apposition, is derived from the Latin noun “elegans”, alluding to the fine, perfect, elegant wing features.

### ﻿Genus Euphlyctinides

Taxon classificationAnimaliaLepidopteraLimacodidae

Hering, 1931

48D87518-5EF1-56E2-9134-3AB7C007F532

Euphlyctinides Hering, 1931: 704. Type species (by original designation): Euphlyctinidesrava Hering, 1931. Type locality: India, Darjeeling.

#### Note.

The genus *Euphlyctinides* was erected by Hering (1931), with the type species, *E.rava* Hering, 1931. The moths in this genus are medium sized, with a yellowish-brown ground colour. The forewings are elongate, with two non-intersecting dark smooth fasciae. The forewing with R_5_ stalked from discal vein near branch R_3_+R_4_. The tibial spurs are 0-2-4. The genus contains four described species to date, two of which are recorded from China (Solovyev 2009; Solovyev and Witt 2009; Wu 2011; Irungbam et al. 2017; Ji 2018).

### ﻿ Euphlyctinides pseudolaika sp. nov.

Taxon classificationAnimaliaLepidopteraLimacodidae

DDAE9D8B-D472-5C2A-93E2-95BDBD1E8981

https://zoobank.org/EAC31D21-0F9B-4E28-A702-F4B93A7A0084

**Figures 7–14.**
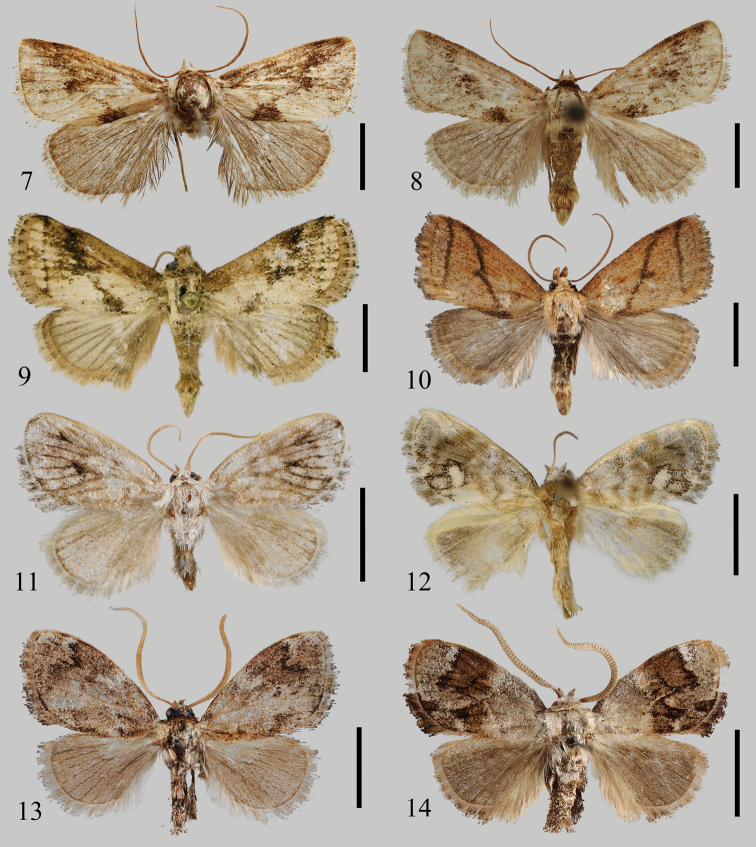
Male adults **7***Euphlyctinidespseudolaika* sp. nov., holotype, Yunnan, China (NEFU) **8***E.pseudolaika* sp. nov., paratype, Yunnan, China (NEFU) **9***E.laika* Solovyev & Witt, 2009, Nghe An, Vietnam **10***E.indi* Solovyev, 2009, Xizang, China (NEFU) **11***Fignyaravalba* sp. nov., holotype, Xizang, China (NEFU) **12***F.melkaya* Solovyev & Witt, 2009, holotype, Lào Cai, Vietnam (MWM/ZSM) **13***Limacocerapachycera* (Hampson, 1897), Xizang, China (NEFU) **14***L.hel* Hering, 1931, Chongqing, China (NEFU). Scale bars: 5 mm.

Figs 7 , 8 , 21 , 22

#### Material examined.

***Holotype*.** ♂, China, Prov. Yunnan, Pu’er City, Manxieba Village, 3.VIII.2018, HL. Han, J. Wu, and MR. Li legs., genit. prep. WuJ-177-1 (NEFU). ***Paratype*.** 1♂, China, Prov. Yunnan, Baoshan City, Mangkuan Village, 30.VII–2.VIII.2014, HL. Han leg., genit. prep. WuJ-702-1 (NEFU).

#### Diagnosis.

The new species is similar in appearance to *E.laika* Solovyev & Witt, 2009 (Fig. 9), but can be separated from the latter by the almost invisible subterminal line, and the weakly sinuous outer margin of the hindwing.

It can also be easily distinguished from the latter by the characters of the male genitalia. In *E.pseudolaika* sp. nov. (Figs 21, 22), the basal flap of the costa in the valva is small, with distinct apical and subapical spines; the juxta is slightly forked apically; the apical process of aedeagus is short and blunt. However, in *E.laika* (Fig. 23), the basal flap of costa is elongate, from the medial part of the valva to its basal, with tiny teeth apically; the apex of juxta is divided into two slender finger-shaped processes; the apical spur of aedeagus is long and acute.

#### Description.

Adult (Figs 7, 8). Forewing length 10.5–11.0 mm, wingspan 23.0–24.5 mm. Head brown; labial palpus brown; antennae filiform, brown. Thorax brown to pale brown. Forewing elongate, ground colour brown, covered with sparse dark scales; anterior basal patch distinct, dark brown; medial fascia sinuous, dark brown, running from ca 2/3 of the costal margin to ca 1/3 of the inner margin from wing base, with large patches on basal, median, and apical area; subterminal line almost invisible; fringe brown. Hindwing reddish brown, with weakly sinuous outer margin; venation distinctly dark brown; fringe brown. Scales on legs brown.

***Male genitalia*** (Figs 21, 22). Uncus elongate, with a strongly sclerotized, acute subapical spur. Gnathos slender, hooked. Valva elongate; base of costa with a distinct flap, which is covered with sparse short spines on the surface and bears a cluster of strongly sclerotized, various-sized, apically acute spines; sacculus slightly inflated at base, lacking sacculus process; cucullus rounded. Juxta flattened, nearly oblong, slightly forked apically. Aedeagus slender, slightly curved near caecum, with a short, blunt apical process coiled in half a turn.

**Female.** Unknown.

#### Bionomics.

The two specimens were collected in late July to early August using a light trap in a coniferous forest; the main vegetation around the collecting site of the holotype consisted of *Pinusyunnanensis* Franch. (Pinaceae) (Fig. 33).

#### Distribution

**(Fig. 29).** China (Yunnan).

#### Etymology.

The name, a noun in apposition, is a combination of the Greek adjective “pseudes” (= false) with the specific name “laika”, showing the similarity with *E.laika*.

#### Remarks.

Although only two males have been collected, the appearance differs from other congeners, particularly in the male genitalia. Hence, in this study, we formally describe them as a new species.

### ﻿ Euphlyctinidis indi

Taxon classificationAnimaliaLepidopteraLimacodidae

Solovyev, 2009

ACAF98F6-F01E-5043-ACCF-3AD7806775B0

Figs 10 , 24

Euphlyctinidis indi Solovyev, 2009: 175. Type locality: Indien WB, Darjeeling Mangpu-road.

#### Material examined.

2♂, China, Xizang Autonomous Region, Linzhi (= Nyingchi) City, Motuo (= Medog) County, Beibeng Countryside, Dergong village, 25.V–4.VI.2021, HL. Han leg., genit. prep. WuJ-552-1 (NEFU); 3♂, China, Xizang Autonomous Region, Linzhi (= Nyingchi) City, Motuo (= Medog) County, Gedang Countryside, 25.V–5.VI.2021, J. Wu and JJ. Fan legs., genit. prep. WuJ-512-2, WuJ-565-1 (NEFU).

#### Diagnosis.

*Euphlyctinidisindi* differs from its congeners by the darker coloration of the forewing, the postmedial line is without distinctive interruptions, the valva is broad in the distal part, and by the juxta without any processes.

#### Bionomics.

The moth flies from May to June. The specimens were collected with a light trap at altitudes of 1,450–2,120 m a.s.l. in a subtropical evergreen broadleaf forest with massive shrubs, ferns, and patches of grassland as in the ground cover layer in the forest (Figs 30, 31).

#### Distribution.

China (Xizang), India.

### ﻿Genus Fignya

Taxon classificationAnimaliaLepidopteraLimacodidae

Solovyev & Witt, 2009

CD0FAD0B-4599-5531-9027-99054953AF0F

Fignya Solovyev & Witt, 2009: 197. Type species (by original designation): Fignyamelkaya Solovyev & Witt, 2009. Type locality: Vietnam, Mt. Fan-si-pan (West).

#### Note.

*Fignya* was first described by Solovyev and Witt (2009). Previously, it contained only the type species *F.melkaya* Solovyev & Witt, 2009, known to be distributed in Vietnam and China. *Fignya* species are small in size, antennae are filiform in both sexes; the labial palpi are slightly up-curved; the forewing has large white spot in the Cu-area with brown border, with sinusoidal vein R_1_, and the veins R_3_+R_4_ are branched from R_5_. The tibial spurs are 0-2-4. In the male genitalia, the gnathos is fishtail-shaped with a comb-like apex; the vesica bears large, strongly sclerotized cornuti (Solovyev and Witt 2009; Ji 2018). The second species of this genus, *F.ravalba* sp. nov., collected from Xizang, is described below.

### ﻿ Fignya ravalba sp. nov.

Taxon classificationAnimaliaLepidopteraLimacodidae

14004841-7DED-5B87-8C43-2ECC46395667

https://zoobank.org/7357A6BD-A186-4503-B97A-1EA60C8C7978

Figs 11 , 25

#### Material examined.

***Holotype*.** ♂, China, Xizang Autonomous Region, Linzhi (= Nyingchi) City, Motuo (= Medog) County, Beibeng Countryside, Dergong Village, 25.V–4.VI.2021, HL. Han leg., genit. prep. WuJ-572-1 (NEFU). ***Paratypes*.** 3♂, China, same data as for holotype, genit. prep. WuJ-573-1, WuJ-556-1, WuJ-557-1 (NEFU).

#### Diagnosis.

The new species is extremely similar to the type species *F.melkaya* (Fig. 12) in appearance, only the ground colour of the whole body is paler than the latter. It can be clearly distinguished from the latter by the male genitalia as follows. In *F.ravalba* sp. nov. (Fig. 25), the basal hairy papula on the valva is small, rounded; the juxta lacks a lateral process; the aedeagus bears several strongly sclerotized long spines at the apical part; the vesica contains two peg-like cornuti. However, in *F.melkaya* (Fig. 26), the basal hairy papula of the valva is larger than in the new species and transverse in shape; the juxta has a pair of slender lateral processes; the apical part of the aedeagus is without any spines; the vesica contains three large hooked cornuti.

**Figures 15–20.**
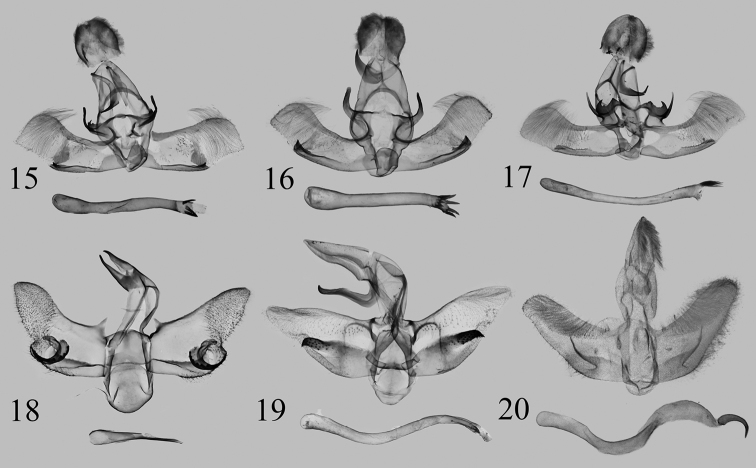
Male genitalia of *Kitanola* spp. **15***K.shilinensis* sp. nov., holotype, Yunnan, China, genit. prep. WuJ-248-1 (NEFU) **16***K.spina* Wu & Fang, 2008, Chongqing, China, genit. prep. WuJ-293-1 (NEFU) **17***K.spinula* Wu & Fang, 2008, Zhejiang, China, genit. prep. WuJ-589-1 (NEFU) **18***K.eleganta* sp. nov., paratype, Xizang, China, genit. prep. WuJ-501-1 (NEFU) **19***K.linea* Wu & Fang, 2008, Guangdong, China, genit. prep. WuJ-610-1 (NEFU) **20***K.uncula* (Staudinger, 1887), Sakhalin, Russia, genit. prep. SAV-10-02 (ZISP).

**Figures 21–28.**
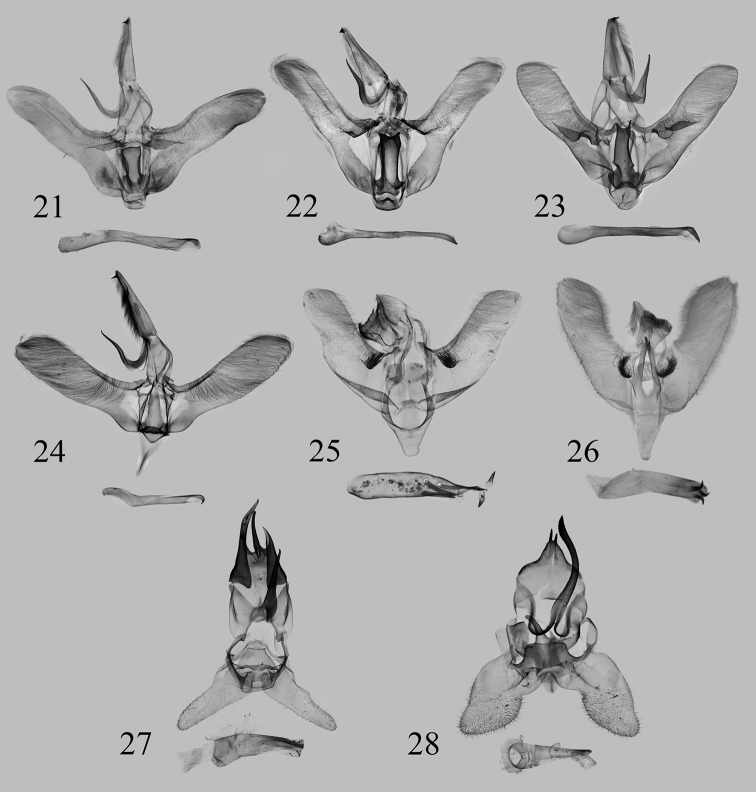
Male genitalia **21***Euphlyctinidespseudolaika* sp. nov., holotype, Yunnan, China, genit. prep. WuJ-177-1 (NEFU) **22***E.pseudolaika* sp. nov., paratype, Yunnan, China, genit. prep. WuJ-702-1 (NEFU) **23***E.laika* Solovyev & Witt, 2009, paratype, Nghe An, Vietnam, genit. prep. 0061 (CASU) **24***E.indi* Solovyev, 2009, Xizang, China, genit. prep. WuJ-552-1 (NEFU) **25***Fignyaravalba* sp. nov., holotype, Xizang, China, genit. prep. WuJ-572-1 (NEFU) **26***F.melkaya* Solovyev & Witt, 2009, holotype, Lào Cai, Vietnam, genit. prep. 14047 (MWM/ZSM) **27***Limacocerapachycera* (Hampson, 1897), Xizang, China, genit. prep. WuJ-555-1 (NEFU) **28***L.hel* Hering, 1931, Chongqing, China, genit. prep. WuJ-287-1 (NEFU).

**Figure 29.**
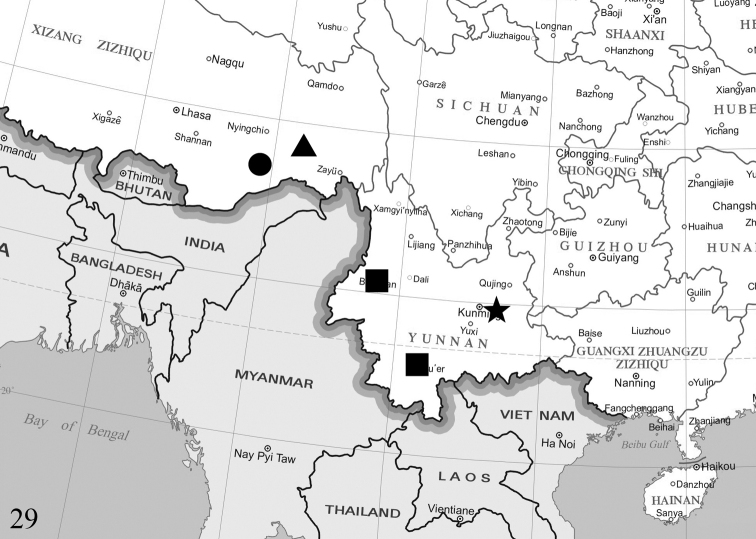
Distribution map of four new Limacodidae species: circle = *Fignyaravalba* (China: Xizang); triangle = *Kitanolaeleganta* (China: Xizang); star = *K.shilinensis* (China: Yunnan); square = *Euphlyctinidespseudolaika* (China: Yunnan).

#### Description.

Adult (Fig. 11). Forewing length 7.0–7.5 mm, wingspan 16–17 mm. Head white; labial palpus white; antennae filiform, pale brown. Thorax with white scales above. Forewing ground colour greyish white, covered with sparse dark scales, a pair of large white spots in Cu-area with brown border; venation visible, brown; fringe dark brown. Hindwing greyish yellow. Abdomen brown to dark brown, mixed with white scales.

***Male genitalia*** (Fig. 25). Uncus pointed apically, without any spur. Gnathos flattened, fishtail-shaped, comb-like apically. Valva elongate, with a basal papula with long bristles; base of sacculus slightly sclerotized; cucullus narrow and rounded. Juxta flattened, weakly sclerotized, without lateral process. Saccus long. Aedeagus short, tube-shaped, thinned proximally, bearing 3–5 strongly sclerotized long spines near apical part; vesica with a pair of strongly sclerotized, peg-like cornuti.

**Female.** Unknown.

#### Bionomics.

**Figures 30–33.**
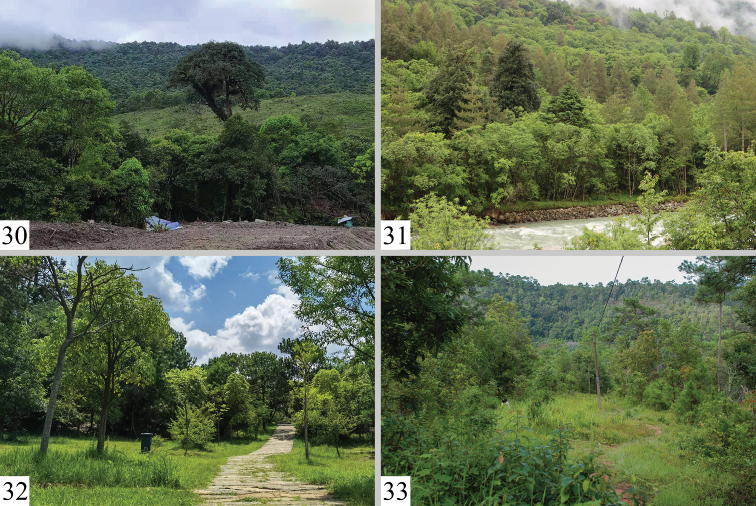
Biotopes of Limacodidae**30, 31** China, Xizang, Motuo County: **30** Beibeng Countryside, Dergong Village, biotope of *Fignyaravalba* sp. nov., *Euphlyctinidisindi* Solovyev, 2009 and *Limacocerapachycera* (Hampson, 1897), photo by HL. Han **31** Gedang Countryside, biotope of *Kitanolaeleganta* sp. nov. and *E.indi*, photographs by J. Wu. **32, 33** China, Prov. Yunnan: **32** Kunming City, Shilin County, Changhu Town, Changhu wetland park, biotope of *K.shilinensis* sp. nov., photo by KL. Wu **33** Pu’er City, Manxieba Village, biotope of *E.pseudolaika* sp. nov., photographs by HL. Han.

The specimens were collected from late May to early June, at an altitude of 1,450 m a.s.l., in a subtropical forest (Fig. 30).

#### Distribution

**(Fig. 29).** China (Xizang).

#### Etymology.

The specific name *ravalba*, an adjective, is derived from the Latin “ravus” (= grey) and “albus” (= white), corresponding to the greyish-white ground colour of the forewing.

### ﻿Genus Limacocera

Taxon classificationAnimaliaLepidopteraLimacodidae

Hering, 1931

480EF4E6-649F-5696-9471-CD4417D63F2B

Limacocera Hering, 1931: 674. Type species (by original designation): Narosapachycera Hampson, 1897. Type locality: India, “Khásis” [Meghalaya, Khasi Hills].

#### Note.

*Limacocera* is a small and rare genus, erected by Hering (1931), with the type species, “*Narosapachycera* Hampson, 1897”. The forewings in this genus are grey, crossed by a characteristic broad, brown medial fascia. The labial palpi are up-curved, almost reaching to the vertex. The base of vein R_1_ in the forewing is strongly curved toward the vein Sc; the vein R_2_ is separate; the veins R_3_+R_4_ are stalked of R_5_. The tibial spurs are 0-2-4. The most obvious feature of this genus are the significantly extended antennae. The female antennae as long as the costal margin of the forewing, whereas the male antennae are longer than the costal margin and markedly enlarged (Hampson 1897; Hering 1931; Holloway 1990; Solovyev and Witt 2009). In China, there was until now only one species known, *L.hel* Hering, 1931, from the type locality in Guangdong Province.

### ﻿ Limacocera pachycera

Taxon classificationAnimaliaLepidopteraLimacodidae

(Hampson, 1897)

33441EC1-80D5-527D-BFF9-C2B2C07638C4

Figs 13 , 27

Narosa pachycera Hampson, 1897: 294. Type locality: India, “Khásis” [Meghalaya, Khasi Hills].
Limacocera pachycera (Hampson): Hering 1931: 674.

#### Material examined.

1♂, China, Xizang Autonomous Region, Linzhi (= Nyingchi) City, Motuo (= Medog) County, Beibeng Countryside, Dergong village, 25.V–4.VI.2021, HL. Han leg., genit. prep. WuJ-555-1 (NEFU).

#### Diagnosis.

The differences between *L.pachycera* (Fig. 13) and its congener *L.hel* (Fig. 14) are that the former is larger than the latter; the dent of the postmedial line incurved above the cell, whereas the same dent in *L.hel* is deeper and incurved below the cell.

The male genitalia of *L.pachycera* (Fig. 27) bear a long, robust, strongly sclerotized uncus; the gnathos is straight and pointed apically; the valva is narrow. However, in *L.hel* (Fig. 28), the uncus is small and divided into two asymmetrical parts; the gnathos is slender, sinuous, and longer than *L.pachycera*; the valva is broad.

#### Bionomics.

The single male specimen was collected in late May to early June at an altitude of 1,450 m a.s.l. in a subtropical forest (Fig. 30).

#### Distribution.

China (Xizang), India.

##### ﻿Checklist of the treated genera with distribution data

*Kitanola* Matsumura, 1925

*K.uncula* (Staudinger, 1887) (China: Heilongjiang; Japan; Korean peninsula; Russia: south-eastern Siberia, Sakhalin)

= *K.sachalinensis* Matsumura, 1925

= *Microcampasuzukii* Matsumura, 1931

= *Microcampacorana* Matsumura, 1931

*K.masayukii* Sasaki, 1998 (Japan)

*K.meridiana* Sasaki, 1998 (Japan)

*K.albigrisea* Wu & Fang, 2008 (China: Shaanxi, Gansu, Henan, Sichuan)

*K.brachygnatha* Wu & Fang, 2008 (China: Yunnan)

*K.caii* Wu & Fang, 2008 (China: Anhui, Henan, Gansu; Japan)

*K.eurygnatha* Wu & Fang, 2008 (China: Zhejiang, Jiangxi, Hunan, Guangdong)

*K.linea* Wu & Fang, 2008 (China: Hubei, Sichuan, Guangxi)

*K.spina* Wu & Fang, 2008 (China: Shaanxi, Sichuan, Chongqing, Hubei, Guizhou)

*K.spinula* Wu & Fang, 2008 (China: Zhejiang, Anhui, Jiangxi, Hunan)

*K.shilinensis* sp. nov. (China: Yunnan)

*K.eleganta* sp. nov. (China: Xizang)

*Euphlyctinides* Hering, 1931

*E.albifusum* (Hampson, 1892) (China: Xizang; India; Bhutan; Nepal)

= *E.rava* Hering, 1931

*E.indi* Solovyev, 2009 (China: Xizang; India)

*E.aeneola* Solovyev, 2009 (China: Yunnan; Thailand)

*E.laika* Solovyev & Witt, 2009 (Vietnam)

*E.pseudolaika* sp. nov. (China: Yunnan)

*Fignya* Solovyev & Witt, 2009

*F.melkaya* Solovyev & Witt, 2009 (China: Sichuan; Vietnam)

*F.ravalba* sp. nov. (China: Xizang)

*Limacocera* Hering, 1931

*L.pachycera* (Hampson, 1897) (China: Xizang; India)

*L.hel* Hering, 1931 (China: Guangdong, Chongqing, Hunan; Vietnam)

## Supplementary Material

XML Treatment for Kitanola

XML Treatment for Kitanola shilinensis

XML Treatment for Kitanola eleganta

XML Treatment for Euphlyctinides

XML Treatment for Euphlyctinides pseudolaika

XML Treatment for Euphlyctinidis indi

XML Treatment for Fignya

XML Treatment for Fignya ravalba

XML Treatment for Limacocera

XML Treatment for Limacocera pachycera
